# Validating child vaccination status in a demographic surveillance system using data from a clinical cohort study: evidence from rural South Africa

**DOI:** 10.1186/1471-2458-11-372

**Published:** 2011-05-23

**Authors:** James Ndirangu, Ruth Bland, Till Bärnighausen, Marie-Louise Newell

**Affiliations:** 1 Africa Centre for Health and Population Studies, University of KwaZulu-Natal, Mtubatuba, South Africa; 2 Glasgow University Medical Faculty, Glasgow, UK; 3 Department of Global Health and Population, Harvard School of Public Health, Boston, USA; 4 MRC Centre of Epidemiology for Child Health, University College London Institute of Child Health, London, UK

## Abstract

**Background:**

Childhood vaccination coverage can be estimated from a range of sources. This study aims to validate vaccination data from a longitudinal population-based demographic surveillance system (DSS) against data from a clinical cohort study.

**Methods:**

The sample includes 821 children in the Vertical Transmission cohort Study (VTS), who were born between December 2001 and April 2005, and were matched to the Africa Centre DSS, in northern KwaZulu-Natal. Vaccination information in the surveillance was collected retrospectively, using standardized questionnaires during bi-annual household visits, when the child was 12 to 23 months of age. DSS vaccination information was based on extraction from a vaccination card or, if the card was not available, on maternal recall. In the VTS, vaccination data was collected at scheduled maternal and child clinic visits when a study nurse administered child vaccinations. We estimated the sensitivity of the surveillance in detecting vaccinations conducted as part of the VTS during these clinic visits.

**Results:**

Vaccination data in matched children in the DSS was based on the vaccination card in about two-thirds of the cases and on maternal recall in about one-third. The sensitivity of the vaccination variables in the surveillance was high for all vaccines based on either information from a South African Road-to-Health (RTH) card (0.94-0.97) or maternal recall (0.94-0.98). Addition of maternal recall to the RTH card information had little effect on the sensitivity of the surveillance variable (0.95-0.97). The estimates of sensitivity did not vary significantly, when we stratified the analyses by maternal antenatal HIV status. Addition of maternal recall of vaccination status of the child to the RTH card information significantly increased the proportion of children known to be vaccinated across all vaccines in the DSS.

**Conclusion:**

Maternal recall performs well in identifying vaccinated children aged 12-23 months (both in HIV-infected and HIV-uninfected mothers), with sensitivity similar to information extracted from vaccination cards. Information based on both maternal recall and vaccination cards should be used if the aim is to use surveillance data to identify children who received a vaccination.

## Background

Child vaccinations are among the most cost-effective public health interventions [1] associated with significant reductions in child mortality [2,3]. Between 2000 and 2007, child deaths from measles declined by an estimated 74% globally and 89% in Africa; polio, a major cause of disability and morbidity among children, is now close to eradication [4]. This success is largely due to the Expanded Programme of Immunization (EPI) which has seen rapid scale-up of routine immunizations; diphtheria-tetanus-pertussis (DTP3) vaccine coverage increased from 20% to 82% and that of measles from 17% to 83% from 1980 to 2008, respectively [5].

Although more children are now protected against vaccine-preventable diseases than ever before, in many settings vaccination coverage is not sufficient to provide herd protection. Measles outbreaks, for instance, continue to occur [6]. For health policymakers to assess the need for vaccination interventions, it is important to monitor coverage at a population level. Two methods to estimate vaccination coverage in young children are commonly used: administrative data, which are unreliable if the target population is poorly enumerated, and may overestimate coverage [7]; and cross-sectional population-based surveys which determine the percentage of children vaccinated within a certain geographic area, such as the demographic and health surveys (DHSs) [8]. DHSs, carried out in over 75 countries, are generally nationally representative and use a two-stage sampling scheme, with clusters as primary sampling units and then random selection of households within each clusters [9]. Vaccination data, collected from the mother (or another household member, if the mother is absent), on all children aged 12-23 months at the time of the interview are used in calculating coverage.

Demographic Surveillance Systems (DSSs), currently totaling 42 in 19 countries, are another source of information on vaccination coverage. DSSs longitudinally track the demographic and health indicators of individuals in well-defined study areas, through household and individual surveys [10]. Finally, clinical cohort studies documenting the vaccination status of children during follow up have also been used to estimate vaccination coverage [11,12]. In contrast to cross-sectional surveys, vaccination data from longitudinal population-based DSSs has never been validated.

This study investigates the sensitivity of vaccination information collected as part of a large, longitudinal, population-based DSS in rural South Africa. Data on vaccinations collected in a large clinical cohort study serves as the "gold standard" in the sensitivity estimation.

## Methods

### Setting

Since 2000, households in the Africa Centre Demographic Surveillance Area (DSA), in the Umkhanyakude district of northern KwaZulu-Natal, South Africa [13,14], have been visited twice yearly as part of a DSS, with questionnaires administered to key household informants recording vital events including vaccinations [14]. HIV prevalence [15], HIV incidence [16], and HIV-related mortality are high in the DSA [17], but mortality has been declining in recent years due to the rapid expansion of antiretroviral treatment [18]. HIV prevalence in women of child-bearing age in the community is particularly high with approximately 40% of pregnant women HIV-infected [19]. In the sub-district, the health service infrastructure includes a central district hospital, 17 fixed primary health care clinics, and 31 mobile clinic points [20]. The mobile clinics offer childhood vaccination in addition to family planning advice and antenatal care. We previously estimated vaccination coverage in the first year of life in the DSA, which was highest for BCG, given at birth, at 89% (95% CI 82-94) and lowest for measles vaccine, given at 9 months, at 77% (95% CI 67-84) [21].

### Survey methods

In the DSA, trained interviewers visited households and administered a standardised questionnaire in the local language, *isiZulu *[14]. Mothers, caregivers, or the head of the household (in absence of the mother or caregiver) were asked to show the interviewers the South African Road-to-Health (RTH) card for all children aged 12-23 months at the time of the surveillance visit. For ease of exposition, we henceforth refer to caregivers and heads of household who provided information on a child's vaccination status as "mother". A child's vaccination history was recorded only once after the child had celebrated their first birthday. The response rates for household surveillance are >99% [14]. The RTH card records dates of all routine vaccinations a child has received [22]. When a child's RTH card was missing, interviewers asked the mother to recall whether the child had received each of the vaccinations included in the South African National Immunization Schedule [23]. Information on the clinic of first vaccination is also recorded. This approach to elicit child vaccination status is similar to that used by DHS [9]. The Africa Centre DSS has a shorter duration of recall than the DHSs; the former collects vaccination histories bi-annually, the latter usually collects these histories every five years although coverage is calculated on children aged 12-23 months. Therefore, in DHSs it may be likely that vaccination cards, especially for older children, are missing and maternal recall will become more important as a tool.

### The Vertical Transmission Study

The Vertical Transmission Study (VTS, 2001-2006) enrolled approximately equal numbers of pregnant, HIV-infected and uninfected women into an intervention cohort study, to examine infant feeding and postnatal HIV transmission [24]. Women and their children had scheduled clinic visits, monthly to 9 months after delivery, and 3-monthly from 10-24 months after delivery. Visits coincided with times for child immunizations; study nurses either performed the immunization themselves or referred the child for immunization to the Department of Health nurse in adjacent clinic rooms. The VTS study nurse then completed the study form appropriately after the immunization had been given. However, if the child had been vaccinated elsewhere, this information was not recorded on the study form. Children with missing vaccination information on the VTS form were classified as 'not vaccinated on scheduled visit' in this study. For these children, we investigated if they were reported as vaccinated in the DSS, as they may have been immunized outside the VTS clinics. This study utilized all the vaccination status information that were either given or observed to have been given by the VTS study nurse. Ethical approval was obtained from the Biomedical Research Ethics Committee (BREC) of the University of KwaZulu-Natal (UKZN).

**Figure 1:**
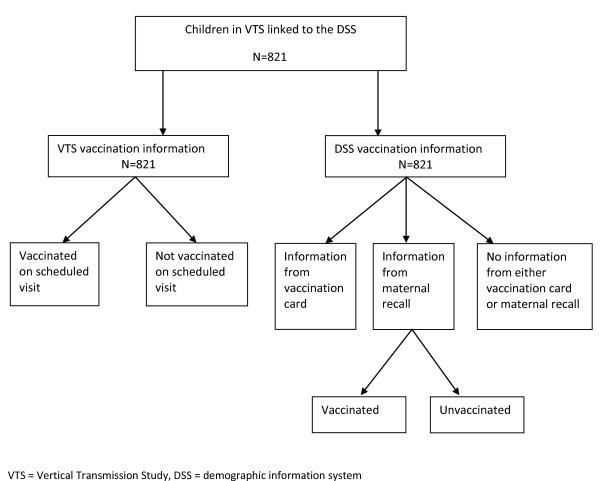
Flow chart of sample selection

The sample includes all children in the VTS who were born in the DSA between December 2001 and April 2005, and linked to the demographic information in the household surveillance of the Africa Centre DSS with follow-up to age 23 months (Figure 1). Only five of the eight VTS recruitment clinics were located in the DSA; and only a proportion of the women enrolled in the VTS lived in the DSA. VTS data were linked to demographic surveillance data in steps; firstly, where perfect match on mother's names, gender, national South African identification number, and date of birth were found in both datasets, the linkage was made automatically. Thereafter 'likely' candidates for linkage, based on less perfect matches, e.g., matching on most but not all criteria, various combinations of similar-sounding names, close dates of birth etc. were made by experienced local staff members. Once all the mothers were matched to the extent possible, a similar exercise was carried out to match the children. Overall, one-third of the mothers and one-third of the children who had, at some point since 2000, been under the surveillance in the Africa Centre surveillance system were successfully matched. For a child to be matched, their mother had to have been already matched. Ethical approval for this linkage was obtained from BREC at UKZN.

In the DSS, vaccination by vaccine type was considered complete as per the South African National Immunization Schedule [23,25] (one vaccine dose of measles; and three doses of poliomyelitis (polio), diphtheria-tetanus-pertussis (DTP), and Hepatitis B (HepB)). Bacillus Calmette-Guérin (BCG) vaccine was excluded because this vaccination was given at birth by midwives and thus not documented on the VTS forms whose first child clinic visit was at 6 weeks of age. *Haemophilus influenzae *type B vaccine (Hib) was also excluded because it was introduced in National Immunization Schedule only in late 2006, i.e., after the first year of life of the last birth in our sample. Mother's report was counted as a vaccination event if the mother stated that the child had received the respective vaccine dose. If the response to the vaccine-dose question was "No", "Don't know" or "Unknown", the child was considered not to have received the particular vaccine dose. This coding of vaccination events follows the DHS guidelines of handling reports of vaccination [9].

### Statistical analysis

We report the distribution of sources of vaccination data among children aged 12-23 months who had received specific vaccinations based on VTS and on DSS information. The 12-23 months age band was chosen because by one year of age, all children should have received all vaccinations included in this study. This age band is commonly used in studies of vaccination coverage [26-28]. We estimated the sensitivity of the DSS in detecting vaccinations conducted as part of the VTS during clinic visits. Sensitivity calculations for RTH card only (from DSS) were based on those children whose RTH card data and VTS data were available; sensitivity calculations for maternal recall only (from DSS) were based on those children whose maternal recall information and VTS data were available. When both RTH card and maternal recall data were available in the DSS, sensitivity calculations were based on all children who had a vaccination report in both DSS and VTS. All analyses were performed in Stata (Version 11: Stata Corporation, College Station, Texas, USA).

## Results

There were 821 children in the VTS with vaccination information, who were linked to the household surveillance (Figure 1). Of these, maternal antenatal HIV status was available for 819 children, of whom 405 (50%) were HIV infected. The median age of the mothers was 23.9 years (IQR 20.2-29.5).

### Vaccination information by data source

Table 1 shows the distribution of vaccination information by data source. The proportion of all children in the VTS who received vaccinations on the scheduled study visit date was highest for Polio1 (86%, 95% confidence interval (CI) 84-88) and lowest for Hepatitis B3 vaccine (48%, 95% CI 45-52).

**Table 1:** Vaccination information by source for children aged 12-23 months (n = 821)

	VTS vaccination information		DSS vaccination information				
**Vaccine**	**Vaccinated on scheduled visit**		**Vaccination card**	**Maternal recall**		**No information from either vaccination card or maternal recall**	
					
	**Yes**	**No**		**Vaccinated**	**Unvaccinated**		

Polio1	708	113	401	210	62	148	
DTP1	708	113	401	208	64	148	
HepB1	702	119	402	201	71	147	
							
Polio2	614	207	405	134	44	238	
DTP2	614	207	403	136	44	238	
HepB2	583	238	404	134	44	239	
							
Polio3	459	362	395	135	45	246	
DTP3	459	362	395	137	44	245	
HepB3	397	424	396	137	45	243	
							
Measles	461	360	361	213	76	171	

The numbers in the cells represent the counts of children with vaccination information from a particular source. VTS = Vertical transmission study, DSS = demographic information system.

In the DSS, 28%, 44% and 45% of infants were vaccinated outside the schedule at 6 (window period 5-7), 10 (9-11) and 14 (13-16) weeks respectively; over 80% of these children received vaccinations within 30 days after the window period. 22% of infants were vaccinated for measles after the age of 9 months (window period 9-10 months), two-thirds of these receiving the vaccine before the age of 2 years. Across all vaccinations, the vaccination data in matched children in the DSS was based on the vaccination card in about two-thirds of the cases and on maternal recall in about one-third. The addition of maternal recall of vaccination status of the child to the RTH card information thus significantly increased the proportion of children known to be vaccinated across all vaccines in the DSS. For instance, the proportion of children known to have received the first dose of the polio vaccine was 49% (95% CI, 45-52) based on vaccination card alone, but 74% (95% CI, 71-77) based on both maternal recall and vaccination card, p < 0.001. The proportion of children known to have received the measles vaccine also increased significantly from 44% (95% CI, 41-47) to 70% (95% CI, 67-73), p < 0.001.

Between 71% and 89% of the children without information for a particular vaccination in the DSS (from either the RTH card or maternal recall) were reported as having received that vaccination in the VTS, suggesting that a large proportion of these children had not previously received the vaccination prior to their VTS scheduled clinic visits. This indicates that the DSS performed well in identifying children who are not vaccinated. Further, across all vaccinations, 55-65% of the children who had not been vaccinated during scheduled visits in the VTS had vaccination information available in the DSS. About 80% of the children in this group had received a particular vaccination, according to the DSS information, indicating that VTS performed well in avoiding duplicate vaccinations in already vaccinated children or that vaccinations missed in the VTS could have been picked up later opportunistically.

### Sensitivity estimation

The sensitivity of the DSS in detecting vaccinations conducted as part of the VTS was high, across all vaccinations, when only information from the RTH card information was used (ranging from 0.94 to 0.97) (Table 2). Maternal recall of vaccination coverage was similarly high across all vaccinations (ranging from 0.94 to 0.98). As both vaccination card data and maternal recall data were separately highly sensitive indicators of coverage of vaccinations done in the VTS, high sensitivities at 0.95-0.97 were noted when the two were combined together (Table 2).

**Table 2:** Sensitivity of vaccination record comparing VTS (gold standard) to DSS vaccination information

	DSS (Card data only)			DSS (Maternal recall data only)			DSS (Card plus maternal recall)		
Vaccination	Sensitivity	95% CI	N	Sensitivity	95% CI	N	Sensitivity	95% CI	N
Polio1	0.97	(0.95-0.98)	395	0.96	(0.91-0.98)	204	0.97	(0.95-0.98)	599
DTP1	0.97	(0.94-0.98)	395	0.98	(0.95-0.99)	201	0.97	(0.95-0.98)	596
HepB1	0.96	(0.94-0.97)	395	0.97	(0.94-0.99)	195	0.96	(0.94-0.97)	590
									
Polio2	0.97	(0.95-0.98)	384	0.97	(0.92-0.99)	128	0.97	(0.95-0.98)	512
DTP2	0.97	(0.95-0.98)	382	0.97	(0.92-0.99)	130	0.97	(0.95-0.98)	512
HepB2	0.97	(0.95-0.98)	384	0.97	(0.94-0.99)	128	0.97	(0.95-0.98)	512
									
Polio3	0.97	(0.94-0.98)	376	0.95	(0.89-0.98)	129	0.96	(0.94-0.98)	505
DTP3	0.96	(0.93-0.98)	376	0.96	(0.90-0.98)	130	0.96	(0.94-0.98)	506
HepB3	0.96	(0.94-0.97)	375	0.94	(0.89-0.98)	131	0.96	(0.94-0.97)	506
									
Measles	0.94	(0.91-0.97)	350	0.96	(0.91-0.98)	191	0.95	(0.92-0.97)	541

The estimates of sensitivity did not vary significantly, when we stratified the analyses by maternal antenatal HIV status. For instance, sensitivity based only on the RTH card data in HIV-infected mothers ranged from 0.95 to 0.97, while it ranged from 0.94 to 0.97 in uninfected mothers. Similarly, using only maternal recall, sensitivities ranged from 0.94-0.98 in both infected and uninfected mothers.

## Discussion

We find high sensitivity of maternal recall of vaccination status in children aged 12-23 months when it is validated against data from an independent cohort study, i.e., A positive vaccination report in the DSS will almost always be correct. We previously showed that in the DSS maternal HIV status was negatively associated with vaccination uptake [21]. In this study, the estimates of sensitivity do not vary significantly by maternal antenatal HIV status, so that it is unlikely that our previous findings resulted from HIV-infected mothers being less likely to correctly report that their children had been vaccinated than HIV-uninfected mothers.

A limitation of the VTS data is that vaccination status was only recorded if a vaccination was given at the scheduled study visit date. Therefore the total proportion of children vaccinated in the study cannot be determined. In this study, we thus merely investigate the sensitivity of DSS-based vaccination information, using vaccinations conducted as part of the VTS as "gold standard". Future studies should estimate the specificity of surveillance-based vaccination records, since for many purposes it is more important to correctly identify children who have not received a vaccination than those who have received it. The coverage rates should be homogenously distributed to avoid pools of unvaccinated children. Thus, in South Africa where routine measles coverage has been static at 76-83% from 1996-2004 [29], and heterogeneously distributed [21], measles outbreaks were reported in 2010, indicating that there is an urgent need to identify children who have not received the measles vaccination [30].

A general limitation of our study design is that we only investigate the sensitivity of information collected as part of a longitudinal demographic surveillance in detecting vaccinations given in a clinical study - i.e., the design does not allow us to directly investigate the sensitivity of the surveillance information in detecting vaccinations given on other dates in other settings, such as antenatal clinics, mobile clinics or general primary healthcare facilities. However, it seems unlikely that the sensitivities of detecting vaccinations given on other dates should be much different from the ones estimated in this study for the VTS vaccinations. First, the process of obtaining information about the VTS vaccinations was the same as the one for a vaccination given in any other setting (extracting information from the RTH card and eliciting maternal recall of vaccinations, if the RTH card was not available). Second, with the exception of mother's VTS participation, all eligibility criteria for children's inclusion in this study were the same as the ones for inclusion in the DSS, in which information on vaccinations given at occasions other than the scheduled VTS visits is available. Finally, the participation rate of eligible mothers in the VTS was extremely high, indicating the study participants are unlikely to differ substantially from the rest of the mothers in the DSS, whose children did not have the opportunity to receive vaccinations through VTS.

The DSS covers the entire population resident in the Africa Centre DSA. Almost all of the households in the DSA participate in the surveillance, i.e., the DSS data are unlikely to suffer from selection biases. While it is possible that the pregnant women who chose to participate in the VTS are not a random sample of all pregnant women in the DSA, it is unlikely that VTS sample was highly selected: almost all pregnant women in the DSA present for antenatal visits [31,32], and the VTS participation rate of antenatal attendees women was >80% [25].

## Conclusions

It is important to accurately estimate vaccination coverage to monitor the performance of the health system [33,34]; to establish the links between vaccination capacity [35], coverage, disease occurrence, and the economic benefits of vaccination [36,37]; and to provide a framework for future coverage goals. Our result that the sensitivity of both maternal recall and information extracted from vaccination cards is near-perfect implies that estimates of vaccination coverage based on either one of these two information sources, or on both sources jointly, are unlikely to be downward biased, increasing our confidence in estimates of vaccination coverage based on DSS and DHS data.

## Competing interests

The authors declare that they have no competing interests.

## Authors' contributions

RB and TB conceived the idea of investigating vaccination in the two studies and participated in analyses and interpretation of results; M-LN supervises both the surveillance and the VTS and contributed to the interpretation of the findings and suggestions for analyses. J.N. was responsible for overall statistical analysis and writing of the paper. TB, RB and M-LN gave constructive comments during the analysis and writing of the paper and all authors substantially contributed to, and approved, the final manuscript.

## Pre-publication history

The pre-publication history for this paper can be accessed here:

http://www.biomedcentral.com/1471-2458/11/372/prepub
